# Unlocking hematopoietic stem cell potential: integrative computational approaches for genomic and transcriptomic analysis

**DOI:** 10.3389/fcell.2025.1589823

**Published:** 2025-09-03

**Authors:** Pawan Kumar Raghav, Basudha Banerjee, Rajni Chadha

**Affiliations:** ^1^ Immunogenetics and Transplantation Laboratory, Department of Surgery, University of California San Francisco, San Francisco, CA, United States; ^2^ BioExIn, Delhi, India

**Keywords:** Hematopoietic stem cells (HSCs), single-cell RNA sequencing (scRNA-Seq), computational biology, regenerative medicine, stem cell therapy, HSC transplantation, self-renewal, differentiation

## Abstract

Hematopoietic stem cells (HSCs) sustain lifelong hematopoiesis through their capacity for self-renewal and multilineage differentiation. However, the isolation and functional characterization of HSCs remain challenging due to their cellular heterogeneity and dynamically regulated transcriptional and epigenetic landscapes. Advances in experimental and computational biology, including single-cell RNA sequencing (scRNA-seq), chromatin immunoprecipitation sequencing (ChIP-seq), network inference algorithms, and machine learning, have improved our ability to resolve transcriptional states, trace lineage trajectories, and reconstruct gene regulatory networks (GRN) at single-cell resolution. These approaches enable the discovery of novel HSC subtypes and regulatory factors, and facilitate the integration of multi-omics data to uncover epigenetic and transcriptional mechanisms that drive stem cell fate decisions. Additionally, machine learning models trained on high-throughput datasets provide predictive power for identifying novel enhancers, transcription factors, and therapeutic targets. This review underscores the synergistic role of computational tools in deciphering HSC biology and highlights their potential to improve stem cell therapies and precision treatments for hematologic disorders.

## 1 Introduction

Hematopoiesis is the process by which hematopoietic stem cells (HSCs) proliferate and differentiate into all blood cell lineages, ensuring the continuous production of blood cells throughout an organism’s life (Ng and Alexander, 2017). HSCs can be sourced from bone marrow, peripheral, and umbilical cord blood (Lee and Hong, 2020). Understanding the regulation of HSC self-renewal and lineage differentiation is crucial for both basic research and clinical applications (Barriga et al., 2012). HSC transplantation remains a cornerstone in treating hematologic malignancies, autoimmune disorders, and immunodeficiencies, where their self-renewal capacity is critical for long-term engraftment and therapeutic success (Weissman and Shizuru, 2008). Despite their substantial clinical utilization, achieving a highly purified HSC population for transplantation continues to pose significant challenges. Standard therapeutic protocols often rely on mobilized peripheral blood or whole bone marrow, which contains a heterogeneous mixture of progenitor and mature cells. Consequently, the proportion of true, self-renewing HSCs is relatively low (Skulimowska et al., 2022). For successful transplantation, an optimal dose of approximately 2 × 10^6^ CD34^+^ cells per kilogram of the recipient’s body weight is recommended (Tricot et al., 2010). However, CD34 expression alone does not guarantee stem cell purity or functional potential. Pharmacological agents like NSC87877, a c-Kit inhibitor, when combined with stem cell factor (SCF), have shown promise for enhancing HSC proliferation post-isolation (Raghav et al., 2018). Increasing the accessibility of highly purified, self-renewing HSCs can enhance therapeutic outcomes and pave the way for novel treatment approaches (Negrin et al., 2000; Logan et al., 2012; Czechowicz and Weissman, 2010).

Computational approaches have emerged as powerful tools to overcome the limitations of HSC identification and characterization by tracing complex gene regulatory interactions (GRN) and epigenetic landscapes that govern HSC fate. Techniques such as single-cell RNA sequencing (scRNA-seq), chromatin immunoprecipitation sequencing (ChIP-seq), network inference algorithms, and machine learning enable the mapping of transcriptional profiles, regulatory networks, and functional heterogeneity at the single-cell level (Kamimoto et al., 2023; Moignard et al., 2015; Wilson et al., 2015; Wang et al., 2024).

Among these technologies, scRNA-seq has proven particularly valuable in revealing transcriptional heterogeneity within HSC populations. It provides high-resolution insights into the lineage commitment and developmental trajectories of HSCs (Wilson et al., 2015; Hérault et al., 2022; Velten et al., 2017). Analytical tools such as FastQC (Andrews, 2010), STAR (Dobin et al., 2013; Du et al., 2020), Seurat (Butler et al., 2018), SCANPY (Wolf et al., 2018), DESeq2 (Love et al., 2014), CellAssign (Zhang et al., 2019), edgeR (Robinson et al., 2010), and Monocle (Trapnell et al., 2014; Qiu et al., 2017a; Qiu et al., 2017b) are commonly used to process and interpret scRNA-seq data. ChIP-seq complements transcriptomic approaches by identifying genome-wide transcription factor (TF) binding sites and epigenetic modifications that regulate HSC self-renewal and differentiation (Cui et al., 2009; Joshi et al., 2013). Tools such as Bowtie2 (Langmead and Salzberg, 2012), MACS2 (Zhang et al., 2008), SICER (Xu et al., 2014), and GREAT (McLean et al., 2010) enable precise mapping of protein-DNA interactions and chromatin dynamics during HSC development. Network inference algorithms are another critical layer in decoding the regulatory circuitry of HSCs. By integrating large-scale expression data, these methods uncover interactions among TFs and their target genes, thereby identifying pivotal regulators such as PU.1, GATA2, LMO2, and MYB (Velten et al., 2017; Wilson et al., 2016; Rodriguez-Fraticelli et al., 2020; Moignard et al., 2013). Tools such as ARACNE (mutual information-based) (Margolin et al., 2006), WGCNA (correlation-based module detection) (Langfelder and Horvath, 2008), Cytoscape (Shannon et al., 2003), and GeneNet (Bayesian network inference) (Ananko et al., 2002) are widely used for inferring and visualizing these networks. Machine learning techniques further enhance our ability to model gene expression, predict regulatory elements, and analyze chromatin accessibility in HSCs (Xiang et al., 2020; Fortelny and Bock, 2020; Lal et al., 2021). Scikit-Learn, DeepCpG, and ChromNet, provide robust data integration, feature selection, model training, and predictive analysis capabilities (Angermueller et al., 2017; Lundberg et al., 2016; Scikit-Learn, 2016; Shannon et al., 2003).

**FIGURE 1:**
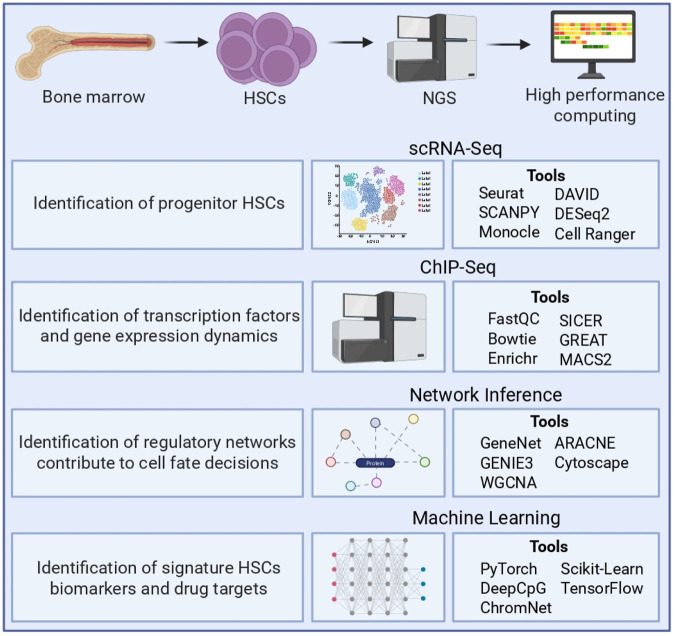
Computational approaches for HSCs’ genomic and transcriptomic data analysis. Illustrates the integrative workflow for analyzing bone marrow-derived HSCs using NGS and high-performance computing. scRNA-seq used to identify progenitor HSCs, resolve transcriptional heterogeneity, and explore cell state transitions using tools such as Seurat, SCANPY, Monocle, DESeq2, DAVID, and CellRanger. ChIP-seq identifies transcription factor binding sites and assesses gene regulatory dynamics, with key tools including FastQC, Bowtie, MACS2, SICER, Enrichr, and GREAT. Network inference approaches, such as GeneNet, GENIE3, WGCNA, ARACNE, and Cytoscape, enable the reconstruction of gene regulatory networks governing HSC fate decisions. Machine learning methods, including PyTorch, DeepCpG, ChromNet, scikit-learn, and TensorFlow, are applied to identify biomarkers, predict regulatory elements, and model gene expression patterns. NGS: next-generation sequencing; scRNA-seq: Single-cell RNA sequencing; ChIP-seq: Chromatin immunoprecipitation sequencing.

Computational approaches revolutionize the understanding of HSC biology by unraveling cellular heterogeneity, elucidating transcriptional and epigenetic control mechanisms, and identifying biomarkers and therapeutic targets. Figure 1 presents a comprehensive framework for unraveling the complexity of HSC regulation by integrating multi-omics data with advanced computational pipelines. This framework ultimately facilitates the isolation and functional validation of pure HSC populations for therapeutic applications.

## 2 Approaches for analyzing HSC genomics and transcriptomic data

Following high-throughput data generation and expression quantification, various computational approaches are employed to analyze genomic and transcriptomic data in HSCs (Figure 1). These methods enable in-depth exploration of transcriptional heterogeneity, regulatory mechanisms, and lineage trajectories. scRNA-seq is a powerful tool for dissecting HSC heterogeneity, allowing the identification of novel cell types, functional states, and regulatory networks (Wilson et al., 2015; Hérault et al., 2022). ChIP-seq reveals genome-wide TF binding sites and epigenetic regulation, including cis-regulatory landscapes of HSCs (Qi et al., 2021). Network inference algorithms use high-throughput expression data to infer regulatory interactions among genes or proteins (Cahan et al., 2021). These approaches have been used to reconstruct transcriptional networks involved in hematopoietic development. Application of network inference on single-cell gene expression data decodes early blood development regulatory programs (Moignard et al., 2015). Machine learning algorithms, including support vector machines (SVMs), random forests, and deep learning, are employed to predict regulatory interactions and identify novel gene networks between endothelial cells and HSCs (Wang et al., 2024).

## 3 scRNA-seq in HSC analysis

scRNA-seq is a technique that enables high-resolution characterization of cellular heterogeneity by profiling gene expression at the single-cell level (Hérault et al., 2022). scRNA-seq has been instrumental in uncovering the transcriptional diversity of HSCs and their progeny. This groundbreaking approach has unveiled novel cell states, differentiation trajectories, and regulatory networks that were previously unknown (Hérault et al., 2022; Ivanova et al., 2002). Seminal studies have utilized scRNA-seq to identify and functionally characterize distinct HSC subpopulations. These studies revealed cells with transcriptional signatures linked to quiescence, immune activation, and a megakaryocyte-erythroid lineage bias (Wilson et al., 2015; Velten et al., 2017; Rothenberg, 2021). The technique has also delved into the differentiation of lineage-specific T and B lymphocytes and has identified transcriptional regulators that commit cells to these lineages (Velten et al., 2017; Rothenberg, 2021). Beyond steady-state hematopoiesis, scRNA-seq has unveiled how radiation affects the transcriptional programs of HSCs, shedding light on stress-induced alterations in quiescence and survival pathways. (Gao et al., 2021; Fast et al., 2021). scRNA-seq offers unprecedented insights into the molecular mechanisms that govern HSC identity and function. (Velten et al., 2017; Rodriguez-Fraticelli et al., 2018). Table 1 outlines the fundamental computational processes involved in analyzing scRNA-seq data and the commonly employed tools for HSC-specific research.

**TABLE 1:** Commonly used computational tools for the analysis of HSCs’ scRNA-seq data. Outlines key analytical approaches, including quality control, normalization, dimensionality reduction, clustering, differential gene expression analysis, pseudotime trajectory inference, and network analysis. For each step, representative tools are listed alongside corresponding references. scRNA-seq, single-cell RNA sequencing; HSCs, Hematopoietic stem cells; PCA, Principal component analysis; t-SNE, t-Distributed Stochastic Neighbor Embedding; UMAP, Uniform Manifold Approximation and Projection; DEGs, Differentially expressed genes; GO, Gene ontology.

Approaches	Steps	Tools	References
Quality control and preprocessing	Cell quality control	FastQC RSeQC	Andrews (2010) Wang et al. (2012)
	Read alignment	STAR HISAT	Dobin et al. (2013) Kim et al. (2015)
	Unique molecular identifier	Cell Ranger Scater	Zheng et al. (2017) McCarthy et al. (2017)
	Gene expression quantification	HTSeq featureCounts	Anders et al. (2015) Liao et al. (2014)
	Quality filtering of cells and genes	Seurat SCANPY	Butler et al. (2018) Wolf et al. (2018)
Normalization	Normalization of sequencing depth	DESeq2 scran	Love et al. (2014) Lun et al. (2016)
	Normalization of gene expression	scran ZINB-WaVE	Lun et al. (2016) Risso et al. (2018)
Dimensionality reduction	PCA	Seurat SCANPY	Butler et al. (2018) Wolf et al. (2018)
	t-SNE	Seurat SCANPY	Butler et al. (2018) Wolf et al. (2018)
	UMAP	UMAP SCANPY	Ghojogh et al. (2021) Wolf et al. (2018)
	Diffusion maps	destiny SCANPY	Angerer et al. (2016) Wolf et al. (2018)
Clustering and cell type identification	Hierarchical clustering	Seurat SCANPY	Butler et al. (2018) Wolf et al. (2018)
	k-means clustering	Seurat SCANPY	Butler et al. (2018) Wolf et al. (2018)
	Density-based clustering	Seurat SCANPY	Butler et al. (2018) Wolf et al. (2018)
	Cluster identification based on marker genes	Seurat CellAssign	Butler et al. (2018) Zhang et al. (2019)
Differential gene expression analysis	DEGs between cell types	DESeq2 edgeR	Love et al. (2014) Robinson et al. (2010)
	GO enrichment analysis	clusterProfiler GSEA	Yu et al. (2012) Subramanian et al. (2005)
Cell trajectory and pseudotime analysis	Ordering of cells along a developmental trajectory	Monocle Slingshot	Trapnell et al. (2014) Street et al. (2018)
	Inference of gene expression dynamics along the trajectory	Monocle scVelo	Trapnell et al. (2014) Bergen et al. (2020)
Network analysis	Construction of gene co-expression networks	WGCNA SCENIC	Langfelder and Horvath (2008) Aibar et al. (2017)

### 3.1 Quality control and preprocessing

scRNA-seq generates high-dimensional raw data that requires extensive preprocessing to ensure analytical accuracy and biological validity (Zheng et al., 2017). This crucial step involves removing technical noise and low-quality cells before downstream analyses. The preprocessing pipeline typically encompasses cell quality assessment, read alignment, unique molecular identifier (UMI) counting, gene expression quantification, and quality filtering.

Cell quality control serves as the foundation and is an essential step for excluding cells with poor-quality reads or abnormal transcript profiles. Tools such as Cell Ranger, Seurat, and RSeQC are being widely used for this purpose (Butler et al., 2018; Zheng et al., 2017; Wang et al., 2012). Cell Ranger evaluates sequencing quality, total read count, and genome-mapping percentages, while Seurat utilizes metrics such as the number of detected genes, UMI counts, and mitochondrial gene content to identify and exclude low-quality cells (Butler et al., 2018).

Read alignment maps sequencing reads to a reference genome. STAR and HISAT are the most commonly used aligners (Dobin et al., 2013; Kim et al., 2015). STAR offers ultrafast and high-accuracy alignment through a two-pass strategy, while HISAT employs hierarchical indexing to efficiently map spliced reads.

UMI counting enables accurate quantification of gene expression by distinguishing between true transcripts (UMIs, short DNA sequences that tag individual mRNA molecules) and PCR duplicates (Smith et al., 2017). Cell Ranger, Drop-seq, and Scater facilitate UMI counting (Macosko et al., 2015; Baran-Gale et al., 2018; McCarthy et al., 2017).

Gene expression quantification typically involves counting UMIs associated with each gene. Commonly used tools include Cell Ranger, HTSeq, featureCounts, and Kallisto (Anders et al., 2015; Du et al., 2020; Zheng et al., 2017; Liao et al., 2014). Cell Ranger quantifies gene expression using the feature-barcode matrix generated from UMI counting (Zheng et al., 2017). Kallisto employs pseudo-alignment for faster transcript quantification without the need for complete read mapping (Bray et al., 2016; Brüning et al., 2022). Quality filtering of cells and genes ensures that only relevant data is retained for downstream analysis. Seurat and Cell Ranger apply user-defined thresholds based on gene detection, UMI counts, and mitochondrial gene expression (Butler et al., 2018; Zheng et al., 2017). Genes expressed in insufficient cells or at extremely low levels are filtered out to minimize noise and enhance statistical power (Wolf et al., 2018). These preprocessing steps are crucial for ensuring the reliability of scRNA-seq analysis. Their extensive validation in HSC studies forms the foundation for robust interpretation of single-cell transcriptomic data.

### 3.2 Normalization

Normalization is a critical step in scRNA-seq data analysis, addressing variability introduced by differences in sequencing depth, capture efficiency, and RNA content across cells (Cuevas-Diaz Duran et al., 2024). Appropriate normalization ensures that observed gene expression differences reflect biological variation rather than technical noise. Several tools have been developed to normalize scRNA-seq workflows, each employing distinct strategies to correct biases.

SCnorm adjusts for cell-specific technical variability using a variance-stabilizing normalization (VSN) approach, allowing accurate comparison of RNA expression levels across cells (Bacher et al., 2017).

Seurat, a widely used R package for scRNA-seq analysis, offers multiple normalization methods. These include global-scaling approaches and Cell Cycle Regression (CCR), which corrects cell cycle-related transcriptional effects that can confound downstream clustering and trajectory analysis (Butler et al., 2018).

DESeq2 is an R package for differential expression analysis, including normalization methods for scRNA-seq data. It uses a model-based approach to estimate size factors that account for differences in sequencing depth across cells (Love et al., 2014).

Other tools, such as scran and ZINB-WaVE, offer alternative frameworks for normalization, particularly for sparse and zero-inflated single-cell datasets (Lun et al., 2016; Risso et al., 2018). The appropriate normalization strategy is essential for accurate differential expression, clustering, and trajectory inference. The choice often depends on the specific characteristics of the dataset and the downstream analytical goals.

### 3.3 Dimensionality reduction

Dimensionality reduction transforms high-dimensional gene expression data into a lower-dimensional space while preserving essential biological variation (Townes et al., 2019). This facilitates data visualization, clustering, and trajectory inference by mitigating noise and computational complexity. Several widely used techniques are applied for HSC scRNA-seq studies.

Principal component analysis (PCA) is a linear method that identifies orthogonal axes (principal components) capturing the variance in gene expression. It is typically the first step in most scRNA-seq workflows and is implemented in tools such as Seurat and SCANPY (Butler et al., 2018; Wolf et al., 2018; Stuart et al., 2019).

t-Distributed stochastic neighbor embedding (t-SNE) is a nonlinear technique that emphasizes local data structure, making it helpful in visualizing distinct cell populations based on expression similarity. It is commonly employed in Seurat and SCANPY for cluster visualization (Butler et al., 2018; Wolf et al., 2018; Stuart et al., 2019).

Uniform manifold approximation and projection (UMAP) is a recent nonlinear method that preserves local and global data structures. UMAP has gained popularity due to its superior scalability and speed over t-SNE, particularly for large datasets. It is supported by SCANPY, and Harmony (Wolf et al., 2018; Korsunsky et al., 2019; Ghojogh et al., 2021).

Diffusion maps model gene expression similarity using diffusion distances, which are robust to noise and particularly useful for capturing continuous trajectories and identifying rare cell states. Destiny and Diffusion Maps are commonly used tools for this purpose (Angerer et al., 2016; Haghverdi et al., 2015).

### 3.4 Clustering

Clustering identifies transcriptionally distinct HSC populations within complex tissues such as the bone marrow (Butler et al., 2018). Multiple clustering strategies have been developed, each with strengths suited to different data structures and biological contexts.

Hierarchical clustering is a widely used method based on the recursive merging of similar cells or genes. The algorithm constructs a dendrogram to represent the nested relationships between clusters, which can then be cut at a desired resolution to define distinct groups. This method is implemented in tools such as Seurat, SCANPY, and Monocle, and has been applied extensively in HSC studies to resolve lineage-specific transcriptional states (Wolf et al., 2018; Qiu et al., 2017a; Satija et al., 2015).

k-means clustering partitions cells into k user-defined clusters by iteratively assigning cells to the nearest centroid and updating centroid positions until convergence. Despite its simplicity, k-means remains effective for well-separated clusters and is supported in frameworks such as Scikit-Learn and Cell Ranger (Scikit-Learn, 2016; Zheng et al., 2017).

Density-based clustering, including Density-Based Spatial Clustering of Applications with Noise (DBSCAN), identifies clusters based on cell density. This method captures clusters of varying shapes and sizes, excluding outliers or rare cell types as noise. DBSCAN is available in Seurat and SCANPY and has been used to delineate heterogeneous populations within HSC datasets (Butler et al., 2018; Wolf et al., 2018; Satija et al., 2015).

Marker gene-based annotation represents a supervised approach that leverages prior knowledge of gene expression signatures specific to known cell types. Tools such as SingleR and CellAssign compare transcriptomes against reference datasets or predefined marker panels to assign cell identities. This approach is particularly valuable for validating cluster annotations or transferring labels across datasets (Zhang et al., 2019; Aran et al., 2019).

### 3.5 Differential expression and enrichment analysis

Differential gene expression (DGE) analysis is a key component of scRNA-seq workflows, enabling the identification of genes that vary significantly across cell types, states, or conditions. DGE analysis has been instrumental in uncovering transcriptional regulators of HSCs associated with differentiation, aging, and lineage commitment (Wang et al., 2019). Several widely adopted tools support DGE analysis in scRNA-seq data.

DESeq2 is an R/Bioconductor package that employs a negative binomial distribution to model count data and estimate dispersion and fold changes between groups (Love et al., 2014). It has been used to identify transcriptional changes in aging HSCs (Adelman et al., 2019).

edgeR is another R/Bioconductor package that similarly models gene expression using a negative binomial distribution and generalized linear models, offering robust statistical frameworks for identifying differentially expressed genes (DEGs) across groups (Robinson et al., 2010). It has been applied in studies investigating dynamic gene expression during HSC differentiation (Lun et al., 2016).

Limma-voom combines linear modeling with precision weights derived from mean-variance relationships in log-transformed count data. This method is effective for scRNA-seq and has been used in both HSC-specific and broader single-cell studies (Lun et al., 2016; Ritchie et al., 2015).

MAST (Model-based Analysis of Single-cell Transcriptomics) utilizes a Bayesian hierarchical framework to model the bimodal distribution of single-cell data. It is particularly suited for zero-inflated datasets and has been widely applied to identify DEGs in HSCs and their progeny (Vanuytsel et al., 2022; Finak et al., 2015).

Initially developed for trajectory analysis, Monocle supports DGE testing along pseudotemporal trajectories, capturing dynamic changes during HSC lineage specification (Trapnell et al., 2014; Gao et al., 2021).

SCDE (Single-Cell Differential Expression) models dropouts and overdispersion using a Bayesian approach and have been applied in studies exploring gene expression dynamics during HSC differentiation (Tusi et al., 2018; Kharchenko et al., 2014).

In parallel with DGE, gene ontology (GO) enrichment analysis is used to interpret biological functions associated with DEG sets, revealing signaling pathways, cellular processes, and transcriptional programs relevant to hematopoiesis. To interpret the biological significance of differentially expressed genes, several widely used tools have been developed for GO enrichment analysis.

DAVID (Database for Annotation, Visualization, and Integrated Discovery), which enables functional annotation of gene lists, has been applied in studies of HSC differentiation (Adelman et al., 2019; Huang et al., 2009).

Enrichr, a web-based tool that offers access to multiple gene set libraries and enrichment algorithms, has been used to identify transcriptional regulators underlying dynamic HSC states (Kuleshov et al., 2016).

GSEA (Gene Set Enrichment Analysis) assesses whether predefined gene sets show statistically significant differences between biological conditions. It has been widely adopted in single-cell studies of HSCs and immune lineages (Subramanian et al., 2005).

ClusterProfiler provides a programmatic interface for GO and pathway enrichment directly within R and supports visualization and statistical comparison of multiple gene sets (Yu et al., 2012; Xu et al., 2024).

These tools have proven essential for decoding the molecular underpinnings of HSC identity and fate decisions. By linking gene expression patterns to functional pathways, DGE and enrichment analyses continue to deepen understanding of the regulatory networks governing hematopoiesis.

### 3.6 Pseudotime analysis

Pseudotime analysis is a computational strategy used to infer the temporal progression of cellular states from static single-cell transcriptomic data. By ordering cells along a putative developmental trajectory based on their gene expression profiles, pseudotime analysis enables the identification of key regulators and pathways involved in differentiation, lineage commitment, and cellular transitions (Street et al., 2018; Bergen et al., 2020; Campbell and Yau, 2019). Several tools have been developed to model pseudotemporal dynamics in HSCs, each using distinct algorithms to reconstruct lineage hierarchies and predict gene expression changes.

Monocle is one of the most widely used tools for pseudotime inference. It employs a reverse graph embedding algorithm to map gene expression dynamics along developmental trajectories. In HSC studies, Monocle has been used to reconstruct differentiation pathways and identify transcriptional regulators of lineage fate decisions (Trapnell et al., 2014; Olsson et al., 2016).

SCORPIUS utilizes a random walk-based algorithm to model the progression of cells along a smooth trajectory, enabling the prediction of future transcriptional states and identifying key regulatory genes. It has been applied to delineate hematopoietic lineage bifurcation, including the transition from HSCs to lymphoid and myeloid progenitors (Liang et al., 2020; Cannoodt et al., 2016).

Wanderlust reconstructs developmental progressions using a minimum spanning tree approach, enabling detailed mapping of sequential gene expression changes. This method has uncovered lineage-specific gene regulatory programs during HSC differentiation (Velten et al., 2017; Bendall et al., 2014).

Waterfall applies a hierarchical clustering framework to model cellular progression, effectively capturing transcriptional transitions and branching events. In HSCs, Waterfall has been used to trace developmental hierarchies and pinpoint regulatory genes involved in early hematopoietic commitment (Shin et al., 2015).

### 3.7 Network analysis

Network analysis provides a systems-level view of gene and protein interactions, enabling the identification of regulatory modules, signaling pathways, and transcriptional hierarchies that govern cellular identity and function (Cahan et al., 2021). In HSCs, network analysis has been pivotal for reconstructing GRN, identifying lineage-specific transcriptional regulators, and uncovering dynamic programs that govern differentiation and stem cell fate decisions. Several computational frameworks have been widely applied to single-cell transcriptomic data for network inference and analysis in HSCs.

Weighted Gene Co-expression Network Analysis (WGCNA) is an R-based package that constructs gene co-expression networks by identifying modules of highly correlated genes. These modules are often associated with biological traits or cell states. WGCNA has been used to identify hub genes and co-expression modules relevant to HSC maintenance and differentiation (Desterke et al., 2020).

SCENIC (Single-Cell Regulatory Network Inference and Clustering) integrates co-expression analysis with motif enrichment to infer TF–target relationships at single-cell resolution (Aibar et al., 2017). In HSCs, SCENIC has enabled the reconstruction of GRN and the identification of lineage-defining TFs and their regulatory targets (Moignard et al., 2015).

Monocle, in addition to trajectory inference, supports dynamic network analysis by modeling gene expression changes over pseudotime. This allows for identifying temporally regulated genes and pathways during hematopoietic differentiation (Trapnell et al., 2014; Olsson et al., 2016).

CellNet is a supervised machine learning tool designed to assess and reconstruct cell type–specific GRN using gene expression data. It has been employed to evaluate the fidelity of engineered or reprogrammed HSCs and to identify regulatory signatures distinguishing distinct hematopoietic states (Cahan et al., 2014; Lu et al., 2016).

Ingenuity Pathway Analysis (IPA) is a commercial platform that maps gene expression data onto curated biological pathways and networks. IPA has been used to identify upstream regulators, canonical pathways, and molecular interactions relevant to HSC signaling and functional specification (Marx-Blümel et al., 2021).

## 4 HSC ChIP-Seq data analysis

ChIP-seq maps genome-wide binding sites of TFs and other regulatory proteins, providing critical insights into the epigenetic regulation of gene expression (Lundberg et al., 2016). ChIP-seq has been instrumental in delineating cis-regulatory landscapes that control self-renewal and lineage commitment of HSCs. The method involves crosslinking DNA and proteins *in situ*, isolating protein–DNA complexes, immunoprecipitating them using target-specific antibodies, and sequencing the recovered DNA fragments. This enables the identification of genomic loci bound by TFs and chromatin-modifying proteins (Gade and Kalvakolanu, 2012). ChIP-seq profiled undifferentiated and activated HSCs to identify dynamic TF binding events and cis-regulatory regions associated with self-renewal and differentiation (Qi et al., 2021). These findings have deepened the understanding of HSC regulation and may inform future therapeutic strategies for hematological diseases.

### 4.1 Computational pipeline for ChIP-Seq data analysis

Computational analysis of ChIP-seq data involves several key steps, each facilitated by specialized bioinformatics tools.

#### 4.1.1 Quality control and preprocessing

Raw sequencing reads must be assessed for quality and trimmed to remove adapters or low-quality bases. FastQC and Trimmomatic tools are routinely used at this stage (Andrews, 2010; Bolger et al., 2014).

#### 4.1.2 Alignment

Cleaned reads are aligned to a reference genome using aligners such as Bowtie2 or BWA, producing binary alignment map (BAM) files that record read locations and mapping quality (Langmead and Salzberg, 2012; Li and Durbin, 2009).

#### 4.1.3 Peak calling

Aligned reads identify enriched regions referred to as “peaks” that signify protein-DNA interactions. Standard tools include MACS2, which models peak significance, and SICER, which is suited for broad enrichment signals (Zhang et al., 2008; Xu et al., 2014).

#### 4.1.4 Peak annotation and functional analysis

Identified peaks are annotated with genomic features (e.g., promoters, enhancers) using ChIPseeker (Yu et al., 2015). Enrichment analysis tools such as GREAT and Enrichr are then used to interpret the functional roles of bound regions (McLean et al., 2010; Kuleshov et al., 2016).

This pipeline enables the discovery of genome-wide TF binding sites, enhancer-promoter interactions, and regulatory motifs central to HSC function.

### 4.2 HSC ChIP-Seq studies

Applying ChIP-seq to HSCs has enabled high-resolution mapping of TF binding sites and chromatin modifications, offering critical insights into the regulatory architecture underlying hematopoiesis (Lundberg et al., 2016; Gade and Kalvakolanu, 2012). Through computational ChIP-seq data analysis, numerous studies have characterized gene regulatory elements that govern HSC self-renewal, quiescence, and lineage specification (Wilson et al., 2016; Cui et al., 2009). A study employed ChIP-seq to map genome-wide TF occupancy in HSCs subpopulations (Subramanian et al., 2023). MACS2 was used for peak calling and HOMER for motif discovery (Zhang et al., 2008) identified dynamic changes in cis-regulatory landscapes during differentiation. The analysis revealed stage-specific binding of key TFs, underscoring the dynamic regulatory programs that orchestrate HSC fate decisions. The distribution of histone modifications H3K4me3 and H3K27me3 in HSCs and their progeny was investigated using Bowtie for read alignment, MACS2 for peak calling, and IGV for visualization (Zhang et al., 2021). The study demonstrated that histone mark distribution is altered during differentiation. These findings suggested that epigenetic reprogramming is pivotal in regulating gene expression and lineage commitment. The function of Polycomb Repressive Complex 2 (PRC2) was examined in HSC regulation (Xie et al., 2014). ChIP-seq profiling of PRC2 components revealed enrichment at genes involved in differentiation. Functional studies showed that loss of PRC2 activity impaired HSC self-renewal and promoted premature differentiation, highlighting its essential role in maintaining stem cell identity. The enhancer landscape during HSC differentiation was characterized by profiling H3K4me1, a histone modification associated with active and primed enhancers. (Lara-Astiaso et al., 2014). The analysis revealed that lineage-specific enhancers are established early and maintained throughout differentiation, serving as epigenetic bookmarks for future transcriptional activation. The study also mapped binding sites of key TFs implicated in lineage choice and functional specification. ChIP-seq delineates the binding profile of GATA1, a master regulator of erythropoiesis, in erythroid progenitors derived from HSCs (Wilson et al., 2016). Bowtie and HOMER demonstrated that GATA1 targets both promoters and enhancers of erythroid-specific genes, reinforcing its central role in erythroid lineage programming. Similarly, ChIP-seq analysis revealed that GATA2, another critical TF in early hematopoiesis, binds to regulatory elements associated with genes essential for HSC maintenance and differentiation (Joshi et al., 2013). Loss of GATA2 disrupted these programs, confirming its indispensable role in sustaining HSC identity.

These studies underscore the power of ChIP-seq to uncover the transcriptional and epigenetic networks that define HSC behavior. High-resolution binding data with advanced computational pipelines facilitate the identification of promoters, enhancers, and TF occupancy patterns that govern key aspects of HSC function from quiescence and self-renewal to lineage commitment (Joshi et al., 2013; Gade and Kalvakolanu, 2012; Hannah et al., 2011). These findings enhance understanding of hematopoietic development and provide a framework for identifying novel targets for therapeutic manipulation in hematological disorders.

## 5 Network inference algorithms

Network inference algorithms offer a robust computational framework for reconstructing GRN from high-throughput gene expression data. These approaches enable the identification of transcriptional regulators, target genes, and functional modules that control cellular processes such as development, differentiation, and lineage commitment (Kamimoto et al., 2023; Mercatelli et al., 2020). Their application has been particularly transformative in the study of HSCs, where understanding the regulatory circuitry is essential for elucidating the mechanisms governing self-renewal, multipotency, and differentiation. Several network inference algorithms have been developed, each with unique strengths and assumptions based on data types and modeling goals (Saint-Antoine and Singh, 2020). These include Bayesian approaches, mutual information-based algorithms, and correlation-based methods. These algorithms have been applied to transcriptomic data, particularly from scRNA-seq, to predict regulatory interactions with increasing granularity and biological relevance. In a study, GENIE3 (tree-based ensemble learning) predicted regulatory interactions and was employed to infer GRN from single-cell expression profiles of developing mouse embryos (Kamimoto et al., 2023). The analysis identified well-established regulators of hematopoiesis, including GATA2, Runx1, and Scl/Tal1, as well as novel candidates such as LMO2 and MYB. Functional validation through genetic perturbation experiments confirmed the predicted regulatory interactions and demonstrated the network’s ability to forecast downstream effects of TF deletion. Similarly, a study used network inference to analyze bulk RNA-seq data from murine HSCs and their progenitors (Cabezas-Wallscheid et al., 2014). Their analysis revealed a GATA2-centered module regulating self-renewal and identified several additional factors involved in HSC lineage priming.

In another study, network inference was applied to human scRNA-seq datasets to reconstruct differentiation trajectories in early hematopoiesis (Velten et al., 2017). The analysis highlighted PU.1 as a key regulator of myeloid lineage commitment, consistent with prior functional evidence. GRN underlying the differentiation of HSCs into all major blood lineages has been reconstructed (Serina Secanechia et al., 2022). Using scRNA-seq data across developmental timepoints identified both canonical regulators (e.g., GATA2, Runx1, Scl/Tal1) and novel contributors such as CEBPα and Spi1. CRISPR-Cas9-mediated perturbations were used to validate predictions, demonstrating the predictive strength of the inferred network. These studies illustrate how integrating expression data with network inference enables mechanistic insights into HSC biology. By revealing both established and previously uncharacterized regulators, these approaches provide a blueprint for understanding hematopoietic fate decisions at a systems level (Armingol et al., 2021).

### 5.1 Computational workflow for network inference in HSCs

The computational reconstruction of GRN in HSCs typically involves four key steps.

#### 5.1.1 Preprocessing

Raw transcriptomic data (e.g., RNA-seq or scRNA-seq) undergo to quality control, normalization, and batch correction to minimize technical variability and retain biological signals (Lun et al., 2016).

#### 5.1.2 Network inference

Preprocessed data are input into network inference algorithms such as GENIE3, ARACNE, WGCNA, and GeneNet. These tools infer edges between TFs and potential targets, constructing initial GRN (Margolin et al., 2006; Langfelder and Horvath, 2008; Ananko et al., 2002).

#### 5.1.3 Network validation

Inferred interactions are validated against known regulatory databases or experimentally using loss-of-function or gain-of-function assays. This step assesses biological plausibility and predictive robustness (Kamimoto et al., 2023).

#### 5.1.4 Network analysis

The final network is analyzed using centrality, modularity, and connectivity metrics to identify master regulators and key subnetworks (Cahan et al., 2021). Tools like Cytoscape is commonly used for visualization and annotation (Shannon et al., 2003).

## 6 Machine learning approaches for HSC data analysis

Machine learning approaches have become indispensable in HSC computational biology, particularly for modeling complex regulatory networks and predicting gene interactions from high-dimensional data. These techniques facilitate the discovery of novel transcriptional programs and molecular mechanisms underlying HSC differentiation, lineage commitment, and self-renewal (Bian and Cahan, 2016). A notable study utilized a deep learning-based framework to predict tissue-specific regulatory interactions between endothelial cells and HSCs using scRNA-seq data from mouse bone marrow (Wang et al., 2024). This approach accurately captured previously unrecognized cross-cell-type interactions, highlighting the capacity of machine learning to elucidate complex intercellular communication.

ChIP-seq data have been integrated with machine learning, including applying a random forest algorithm to predict TF binding sites, identifying key regulators of HSC function and differentiation (Kamimoto et al., 2023). This highlights the utility of machine learning for enhancer and TF motif prediction. SVMs have also been applied to classify distinct stages of HSC differentiation based on gene expression profiles. A study delineated hematopoietic progenitor cell phenotyping through machine learning approaches, offering insights into the transcriptional differences from fetal liver HSCs (Fidanza et al., 2020). A neural network model has been developed to identify functional enhancers regulating self-renewal and lineage-specific regulators (Xia et al., 2020), and machine learning has also been used to estimate the regulatory potential of DNA sequences, identifying transcription factors and enhancer elements relevant to HSC identity and fate (Xiang et al., 2020).

Other studies have demonstrated the predictive power of random forest models in modeling gene expression changes during HSC differentiation and mapping chromatin accessibility across regulatory regions (Fortelny and Bock, 2020; Lal et al., 2021). Collectively, these applications underscore the transformative role of machine learning in decoding regulatory complexity in HSC biology (Fidanza et al., 2020).

### 6.1 Machine learning tools for HSC data analysis

Several computational tools and platforms have been developed to implement machine learning techniques for HSC datasets.

#### 6.1.1 Scikit-learn

A widely used Python library offering an extensive suite of machine learning algorithms, including SVM, decision trees, and clustering. It has been applied in studies predicting intercellular regulatory interactions (Wang et al., 2024; Scikit-Learn, 2016).

#### 6.1.2 TensorFlow

A robust open-source framework developed by Google, suitable for large-scale deep learning applications. TensorFlow constructs a neural network model to predict gene expression in single HSCs (Athanasiadis et al., 2017).

#### 6.1.3 PyTorch

An alternative deep learning platform known for its flexibility and dynamic computation graph, used to model lineage trajectories of individual HSCs (Wang et al., 2024).

#### 6.1.4 DeepCpG

A deep learning model for predicting DNA methylation from sequencing data. It has been used to model methylation dynamics at single CpG resolution in HSCs and progenitors (Angermueller et al., 2017).

#### 6.1.5 ChromNet

A tool that infers chromatin interactions from ChIP-seq data using deep learning, applied to predict enhancer-promoter connectivity in HSCs (Lundberg et al., 2016).

### 6.2 Machine learning based workflow for HSC data analysis

Machine learning-driven analysis of HSC data typically follows a structured workflow.

#### 6.2.1 Data preprocessing

Raw expression or epigenomic data are filtered, normalized, and batch corrected. Genes with low expression or limited variance are excluded (Gonzalez Zelaya, 2019).

#### 6.2.2 Feature selection

Informative features are extracted to reduce dimensionality and improve model generalizability. Approaches such as minimum redundancy maximum relevance (mRMR) are commonly employed (Dhal and Azad, 2022).

#### 6.2.3 Model training

Selected features are used to train machine learning models such as SVM, random forests, or neural networks (Bian and Cahan, 2016).

#### 6.2.4 Model evaluation

Cross-validation or independent test sets assess model performance, ensuring robustness and avoiding overfitting (Xiong et al., 2020).

#### 6.2.5 Network analysis

Predicted regulatory interactions are visualized and interpreted using platforms like Cytoscape, aiding in identifying key regulators and pathways (Shannon et al., 2003).

### 6.3 Case study: regulatory prediction between endothelial cells and HSCs

A complete machine learning pipeline was demonstrated in a study aimed at decoding HSCs based on their morphological features, using microscopy images, enabling rapid identification of HSCs and progenitor cells (Wang et al., 2024). The SVM model was trained and validated using cross-validation techniques after applying a mutual information-based minimum redundancy maximum relevance algorithm for feature selection (Dhal and Azad, 2022). The resulting network, visualized using Cytoscape, revealed novel intercellular signaling pathways that were experimentally supported, showcasing the strength of machine learning for hypothesis generation and network reconstruction.

## 7 Conclusion

HSC biology has entered a transformative era, driven by advances in high-throughput sequencing technologies and the parallel development of sophisticated computational frameworks. From scRNA-seq and ChIP-seq to network inference algorithms and machine learning, these techniques and tools have collectively revolutionized our ability to dissect the heterogeneity of HSCs, trace lineage trajectories, and decipher regulatory circuits at unprecedented resolution. Crucially, computational strategies enhance the identification and functional characterization of true, self-renewing HSCs. They also facilitate the discovery of biomarkers, transcriptional regulators, and epigenetic modifiers that underpin hematopoietic differentiation. Integrating multi-omics datasets with predictive modeling and functional validation is poised to unlock deeper mechanistic insights into normal and pathological hematopoiesis. The convergence of machine learning, systems biology, and experimental hematology will be essential for achieving the long-standing goal of prospectively isolating and therapeutically deploying pure HSC populations. Furthermore, linking these computational insights to clinical outcomes can accelerate the development of precision therapies for hematologic malignancies, bone marrow failure syndromes, and immune disorders. In essence, computational approaches are no longer ancillary tools in HSC research; they are central to the next-generation of discoveries and therapeutic innovations in stem cell biology and regenerative medicine.

## Abbreviations

BAM, Binary Alignment Map; CCR, Cell Cycle Regression; ChIP-Seq, Chromatin Immunoprecipitation Sequencing; DAVID, Database for Annotation, Visualization, and Integrated Discovery; DBSCAN, Density-Based Spatial Clustering of Applications with Noise; DEGs, Differentially Expressed Genes; DGE, Differential Gene Expression; GO, Gene Ontology; GRN, Gene Regulatory Network; GSEA, Gene Set Enrichment Analysis; HSCs, Hematopoietic Stem Cells; IPA, Ingenuity Pathway Analysis; MAST, Model-based Analysis of Single-cell Transcriptomics; NGS, Next-Generation Sequencing; PCA, Principal Component Analysis; PRC2, Polycomb Repressive Complex 2; SCDE, Single-Cell Differential Expression; SCF, Stem Cell Factor; scRNA-Seq, Single-cell RNA-Sequencing; SVM, Support Vector Machines; TFs, Transcription Factors; t-SNE, t-Distributed Stochastic Neighbor Embedding; UMAP, Uniform Manifold Approximation and Projection; UMIS, Unique Molecular Identifiers; VSN, Variance-Stabilizing Normalization; WGCNA, Weighted Gene Co-expression Network Analysis.
